# Emotional difficulties in older patients with hemodialysis treatment - decision opportunity

**DOI:** 10.1192/j.eurpsy.2023.1858

**Published:** 2023-07-19

**Authors:** M. Klaric, V. Klaric, D. Klaric

**Affiliations:** 1 Faculty of Medicine, University of Rijeka, Rijeka; 2 Department of Psychiatry; 3 Department of Dialysis, Zadar General Hospital, Zadar, Croatia

## Abstract

**Introduction:**

Rapid increase in the number of elderly patients in the world who need dialysis treatment. High age with dementio depression and anxiety negatively affects the outcome of hemodyalisis patients on (HD).

**Objectives:**

The goal is that patients get the ability to choose by themselves will they start HD treatment or not. The old population is often exposed doctors, family or guardian’s descisions that are against their wishes, either due to a lack of communication or lack of knowledge of working methods and procedures. Procedures can leave mental and physical consequences (suffering), no matter they were all done professionally.

**Methods:**

We analyzed old (70-75 years) and very old patients (over 80 years). Cross-sectional analysis of survival of patients at the Hemodialysis (HD) Center with standard methods.

**Results:**

For patients who started hemodialysis at the age of 70 or more, the average survival was: 20.27±18.62 months, those who died 15.54±17.35, and living ones 30.29±17.85. 35% of the patients survived up to one year, two years 18%, and 3 or more 8%.

Most of old people that started dialysis treatment afterwards complained and concluded that it wasn’t necessary mostly because of the lack of communication, or simply it wasn’t their own decision.

Based on these facts, and knowing that these procedures can leave mental and physical consequences subjective assessment about starting dialysis treatment should respect, in the first place, patient’s decision.

The plan for medical procedure involves great ethical, legal and psychological engagment. It often occurs in people who, for some reason, are not in contact and do not adequately test reality.

**Conclusions:**

We are of the opinion that it is, therefore, important that every person, while in mental, physical and social well-being, makes a decision about his medical treatment and communicates it to his family, but it is also very difficult because it touches on his own mortality and helplessness.

**Disclosure of Interest:**

None Declared

